# Progress in Ophthalmology

**Published:** 1903-04-04

**Authors:** 


### PROGRESS IN OPHTHALMOLOGY.

**Contagious Ophthalmia in industrial schools and asylums.**—Dr. Derby [1] in a paper on this subject points out that in 1886 the New York Academy of Medicine appointed a committee to investigate and report on the subject of contagious eye diseases in the asylums and residential schools of the city. They visited the 50 and more asylums of New York, they found that one out of every four children had contagious eye disease. While there were asylums where but few cases existed, there were others in which more than half of the inmates had eye disease of a severe and dangerous type, and where, as Dr. Roosa reported, "It is a marvel to me that there is a healthy eye in the establishment." After an analysis of these various reports it was found that in many of these asylums the eyes of the children were not examined before admission, and that those who were known to have eye disease were not quarantined. In a common dormitory, lavatory, and living-room there were found children with pronounced ophthalmia in daily contact with those whose eyes were as yet unaffected. The children were all washed in one lavatory where a group of 18 were provided with two or three towels per day. The committee received reports from 51 institutions, containing 12,684 inmates; of these 3,862 were declared to have contagious ophthalmia. The committee was satisfied that legislation was necessary, and to that end an Act entitled, An Act for the Better Preservation of the Health of Children in Institutions, was drawn up. This Act provided that each child should be examined before admission. That no child suffering from contagious disease should be allowed to enter or remain in any institution in contact with children not so affected, but should be immediately isolated or placed in a proper room or infirmary provided for that purpose. It provided against the overcrowding of dormitories. It is made incumbent on the physician of each institution to render to the local board of health a monthly report, declaring therein all cases of contagious disease whatever, that existed among the children under his charge. This Act was passed by the Legislature on June 14th, 1886. With the passage of the Act the committee appointed by the Academy of Medicine was discharged, and now after 15 years' operation of the law it is possible to see some of the results. Cases of ophthalmia which were in 1886 roughly 30 per cent., are now in 1903 reduced to roughly 4 per cent. The diminution is most remarkable, and it is instructive to see how this diminution is mainly brought about. Two schools are sufficient to show the working; in each of these the children on admission are examined and immediately isolated if they exhibit contagious eye troubles. A building has been set apart for isolation, and contains schoolroom, work-rooms, sleeping rooms, shower baths, etc. Isolation is absolute till the cases are cured, then they are sent to the main department, where they wash apart from the others, and are inspected tri-weekly for fear of a relapse, in which event they are at once returned to the eye pavilion. Each child at the institution has its individual towel and jets of water, no basins or bath tubs being used. The linen and towels of the contagious pavilion are disinfected and washed separately. Each month the eyelids of each inmate are everted and examined as required by law. In conclusion, he quotes from the report of the superintendent of the New York Blind Asylum, which states that, the number of children in that institution at the close of the school year of 1898 was 169, a reduction since the end of the school year of 1886 of 47 pupils, and this reduction for the last 13 years is really more considerable than it appears when one takes into consideration the growth of the city population in those years. One would expect that in New York where there is a dense population the proportion of eye diseases would be greater than in the country districts; but this is not so. The figures taken from the last census show the number of blind in the county of New York to be 1 in every 2,500 of population, while from five rural counties the number is 1 to each 650 of population, a striking comparison, showing the improvement one may expect in the future.

**Adrenalin in Eye Diseases.**—Sydney Stephenson [2] says of this remedy that it is useful two ways—(1) Before operations, and (2) in certain diseases of the eye. He recommends the following solution, which is to be instilled three or four times in the conjunctival sac before any cutting operation takes place as squint or cataract extraction: Cocain hydrochlor. gr. viii., Adrenalin chloride (1–2,000 sol). ℥iv. The use of the combined solution prevents bleeding, and even renders oozing uncommon. It has been said, perhaps with truth, that its employment may be followed later by reactionary hæmorrhage which may assume alarming proportions. Stephenson says he has had no personal experience of such a disaster, but lately we have had very free hæmorrhage in two operations where it was used, viz., an old blind glaucomatous eye which was removed for the relief of pain, and which bled very freely and for a long time after the operation; and in a case of iridectomy also for an old glaucoma where the anterior chamber and part of the vitreous chamber also was filled with blood—of course, one is used now and then to hæmorrhage in glaucoma operations—but in both these cases the bleeding was quite out of the common. The diseases in which Stephenson thinks adrenalin has rendered much service are phlyctenular conjunctivitis and keratitis, iritis, interstitial keratitis, spring catarrh, and glaucoma. He found it especially valuable in large and vascular conjunctival phlyctenulæ, which under its influence were often cured in two or three days; but then again we have many times seen these large phlyctenulæ clear up after dusting with calomel, which seemed to act like a charm. In iritis he employs atropin sulphate gr. i., adrenalin chloride (1-2000) ℥iv. The same formula may be used three or four times a day, in the earlier initiative stages of interstitial keratitis along with specific treatment by mercurials, preferably injected subcutaneously, or rather intramuscularly. In glaucoma of chronic type he combines physostigmin or pilocarpin or arecolin with the 1-2000 adrenalin chloride solution.

[1] Med. Rec., July 5, 1902. [2] Merck's Archives, Aug. 1902.

